# Prognostic Implication of Heart Failure With Reduced and Midrange Ejection Fraction in Adults With Congenital Heart Disease

**DOI:** 10.1016/j.cjcpc.2026.02.006

**Published:** 2026-03-04

**Authors:** Mohamed Ellabbad, Ahmed Bahnasy, Meena Bai, Sara Aboelmaaty, Ahmed E. Ali, William R. Miranda, Heidi M. Connolly, Alexander C. Egbe

**Affiliations:** Department of Cardiovascular Medicine, Mayo Clinic, Rochester, Minnesota, USA

**Keywords:** heart failure, mortality, prognostication

## Abstract

**Background:**

In the acquired heart disease population, heart failure (HF) is broadly classified based on left ventricular (LV) ejection fraction (EF) as HF with reduced EF (HFrEF), HF with midrange EF (HFmrEF), and HF with preserved EF (HFpEF). The prognostic role of this LVEF-based HF classification has not been evaluated in the adult congenital heart disease (CHD) population because of anatomic and physiological heterogeneity within this population. The current study aimed to address this knowledge gap, by assessing the prevalence, predictors (correlates), and prognostic implications of HFrEF in adults with CHD.

**Methods:**

This is a retrospective cohort study of adults with CHD and morphologic LV as the systemic ventricle. Patients were classified as HFrEF (LVEF ≤40%), HFmrEF (LVEF 41%-49%), and pEF (LVEF ≥50%).

**Results:**

Of 7981 patients, 7203 (90%) had pEF, 451 (6%) had HFmrEF, and 327 (4%) had HFrEF. The correlates of HFrEF were older age, male sex, pacemaker implantation, multiple prior sternotomies, and acquired comorbidities such as hypertension, coronary artery disease, diabetes, and atrial fibrillation. The 15-year mortality risk was 18%, 36%, and 67% in the pEF, HFmrEF, and HFrEF groups, respectively (*P* < 0.001). Both HFmrEF (adjusted hazard ratio: 1.64 [95% confidence interval: 1.29; 2.09], *P* < 0.001) and HFrEF (adjusted hazard ratio: 3.33 [95% confidence interval: 2.72; 4.27], *P* < 0.001) were associated with mortality on multivariable analysis.

**Conclusions:**

Given the elevated risk of mortality in patients with LV systolic dysfunction (both mrEF and rEF) and the emerging data suggesting the efficacy of HF therapy in patients with CHD, there is an urgent need to implement HF therapy in this population.

## Résumé

**Contexte:**

Dans la population atteinte d’une cardiopathie acquise, l’insuffisance cardiaque (IC) est généralement catégorisée en fonction de la fraction d’éjection ventriculaire gauche (FEVG), soit en IC avec FE réduite (ICFEr), en IC avec FE moyenne (ICFEm) ou en IC avec FE préservée (ICFEp). Le rôle pronostique de cette classification de l’IC en fonction de la FEVG n’a pas été évalué dans la population adulte atteinte d’une cardiopathie congénitale, compte tenu de l’hétérogénéité anatomique et physiologique qui caractérise cette population. La présente étude visait à combler cette lacune en évaluant la prévalence, les facteurs de prédiction (corrélats) et la signification pronostique de l’ICFEr chez les adultes atteints d’une cardiopathie congénitale.

**Méthodologie:**

Il s’agit d’une étude de cohorte rétrospective menée chez des adultes atteints d’une cardiopathie congénitale chez qui le ventricule gauche morphologique tient lieu de ventricule systémique. Les patients ont été classés comme suit : ICFEr (FEVG ≤ 40 %), ICFEm (FEVG entre 41 % et 49 %) et FEp (FEVG ≥ 50 %).

**Résultats:**

Sur 7 981 patients, 7 203 (90 %) présentaient une FEp, 451 (6 %) une ICFEm et 327 (4 %), une ICFEr. Les corrélats de l’ICFEr étaient l’âge avancé, le sexe masculin, l’implantation d’un stimulateur cardiaque, les antécédents de sternotomies multiples et les affections concomitantes acquises, comme l’hypertension, les coronaropathies, le diabète et la fibrillation auriculaire. Le risque de mortalité à 15 ans était respectivement de 18 %, de 36 % et de 67 % dans les groupes FEp, ICFEm et ICFEr (*p* < 0,001). L’ICFEm (rapport des risques instantanés [RRI] ajusté : 1,64; intervalle de confiance [IC] à 95 % : 1,29 à 2,09; *p* < 0,001) et l’ICFEr (RRI ajusté : 3,33; IC à 95 % : 2,72 à 4,27; *p* < 0,001) ont été associées à la mortalité dans une analyse multivariée.

**Conclusions:**

Le risque élevé de mortalité chez les patients atteints d’une dysfonction systolique du VG (FEm et FEr) et les données récentes à l’appui de l’efficacité du traitement de l’IC chez les patients atteints d’une cardiopathie congénitale montrent combien il est urgent de traiter l’IC dans cette population.

Heart failure (HF) is the leading cause of mortality in adults with congenital heart disease (CHD).^1^, ^2^, ^3^, ^4^, ^5^, ^6^, ^7^, ^8^ The etiology of HF in CHD is multifactorial and includes valvular disease, ventricular dyssynchrony from rhythm abnormalities, arterial dysfunction (both pulmonary and aortic), and acquired comorbidities.^9^, ^10^, ^11^ As a result of these differences in the disease pathogenesis, defining the prevalence of, risk factors for, and prognostic implications of HF in adults with CHD remains challenging.

In the acquired heart disease population, HF is broadly classified based on left ventricular (LV) ejection fraction (EF) as HF with reduced EF (HFrEF), HF with midrange EF (HFmrEF), and HF with preserved EF (HFpEF).^12^ The treatment options, response to therapy, and natural history differ between these LVEF-based HF categories and provide the foundation for the guideline-directed medical therapy (GDMT) recommendations in this population.^4^^,^^6^^,^^12^ Considering the impact of HF in the natural history of patients with CHD, there is a need to delineate the pathophysiology of HF in CHD, and how this compares with HF in the acquired heart disease population. This will provide the foundation for determining appropriate standard GDMT recommendations. It will also be important to determine whether GDMT used in acquired heart disease, supported by robust empirical data, translates to the CHD population. The purpose of this study was to provide this foundation. We aimed to determine the prevalence, predictors (correlates), and prognostic implications of HFrEF in adults with CHD.

## Methods

### Study population

This is a retrospective cohort study of adults (aged ≥18 years) with CHD biventricular circulation and the morphologic LV as the systemic ventricle who received care at Mayo Clinic from January 1, 2003, to December 31, 2023. We excluded patients with Fontan physiology, congenitally corrected transposition of great arteries, d-transposition of great arteries status post atrial switch operation, and unrepaired/palliated cyanotic CHD. From this cohort, we identified patients with a current or prior history of HF and LVEF <40% (HFrEF) and those with a current or prior history of HF and LVEF 40%-49% (HFmrEF) at the first outpatient evaluation in the adult CHD clinic after January 1, 2003 (baseline encounter). The patients with LVEF ≥50% regardless of the history of HF were classified as the pEF group.

### Data collection

#### Echocardiography

Comprehensive 2-dimensional, Doppler, and speckle tracking echocardiography was performed according to contemporary guidelines.^13^^,^^14^ Offline image analyses were performed by research sonographers using a standardized protocol in the Mayo Adult Congenital Heart Disease Registry Imaging Core Laboratory.^6^^,^^15^^,^^16^ LV volumes and LVEF were assessed using the biplane Simpson method.^13^ In addition, LV global longitudinal strain was assessed by speckle tracking echocardiography using standard techniques as previously described.^13^^,^^14^ Similarly, chamber (right ventricular, right atrial, and left atrial) size and function were assessed by 2-dimensional and speckle tracking echocardiography, and the severity of valve regurgitation was assessed by qualitative Doppler echocardiography.^13^^,^^14^

#### Other study variables

Cardiac magnetic resonance imaging and cardiac catheterization procedures performed within 12 months from the baseline encounter were reviewed, and hemodynamic indices were retrieved from the procedure reports. Similarly, laboratory data including N-terminal pro–B-type natriuretic peptide (NT-proBNP) were retrieved from the medical records.

#### Study definitions

In addition, clinic notes and surgical reports were reviewed to determine CHD diagnosis, and these CHD diagnoses were classified as simple, moderate, or complex lesion based on the criteria from the guidelines for the management of adults with CHD (Supplemental Table S1).^17^ Supplemental Table S2 shows the underlying CHD diagnoses of the patients in the study.

For the purpose of this study, we defined isolated right heart disease as patients with CHD diagnoses involving only the right heart, without concomitant left heart lesions (mitral valve disease, aortic valve disease, or coarctation of aorta).^4^ We defined LV outflow tract (LVOT) disease as the presence of any of the following conditions: aortic valve prosthesis; ≥moderate subvalvular, valvular, or supravalvular aortic stenosis; or ≥moderate aortic regurgitation, in the absence of mitral valve disease.^18^ In patients with LV systolic dysfunction, the etiology was presumed to be ischemic if the patients had a diagnosis of coronary artery disease. Coronary artery disease was defined as myocardial infarction, stable and unstable angina, prior coronary arterial revascularization, or greater than 50% stenosis of a major epicardial coronary artery based on angiography.^19^

### Statistical analysis

Data were presented as mean ± standard deviation, median (Q1; Q3), and count (%). Between-group comparisons were performed using the analysis of variance test, Wilcoxon rank sum test, unpaired *t* test, and Fisher exact test, as appropriate. The predictors (correlates) of HFrEF were assessed using logistic regression analysis. The variables included in the univariable logistic regression models were selected based on clinical relevance, and the covariates with *P* < 0.1 on univariable analysis were used to create the multivariable model. The final covariate selection was based on stepwise backward selection with *P* < 0.1 as the criterion for a variable to remain in the multivariable model.

All-cause mortality was assessed as time-to-event outcome, and ascertained from baseline echocardiogram to outcome, last clinical encounter, or December 31, 2023, and was ascertained by review of medical records and Accurint mortality database. Cox regression analysis was used to assess the relationship between LVEF-based HF category and all-cause mortality. Similar to the methods used for the logistic regression models, we created univariable Cox models using demographic indices, surgical indices, comorbidities, laboratory data, and echocardiographic indices. These covariates were then used to create the multivariable model. Subgroup analyses were performed in patients with NT-proBNP data (N = 3838) and patients with cardiac catheterization data (N = 1710). The cumulative incidence of all-cause mortality was assessed using the Kaplan-Meier method and compared using the log-rank test.

All statistical analyses were performed with BlueSky Statistics software (version 7.10; BlueSky Statistics LLC, Chicago, IL) and JMP statistical software (version 17.1.0; JMP Statistical Discovery LLC, Cary, NC). A *P* value of <0.05 was considered to be statistically significant for all analyses.

## Results

### Baseline characteristics

Of 8207 patients who met the study inclusion criteria, offline assessment of LVEF was feasible in 7981 (97%) patients, and these patients comprised the study group. Of the 7981 patients, 7203 (90%) had pEF, 451 (6%) had HFmrEF, and 327 (4%) had HFrEF. Table 1 compares the baseline characteristics across the 3 groups. Compared with the pEF group, patients in the HFmrEF and HFrEF groups were older, more likely to be male, and more likely to have complex CHD, LVOT disease, prior surgical interventions, comorbidities, and end-organ dysfunction (Table 1).

**Table 1:** Baseline characteristics

	pEF (N = 7203, 90%)	HFmrEF (N = 451, 6%)	HFrEF (N = 327, 4%)	*P*
Demographic indices				
Age (y)	39 ± 16	43 ± 17∗	46 ± 16∗	<0.001
Male sex	3390 (47)	277 (61)	203 (62)	<0.001
Body mass index (kg/m^2^)	27.2 ± 7.1	26.7 ± 5.6	27.9 ± 7.3	0.22
Functional and aerobic capacity				
Peak VO_2_ (mL/kg/min)	25 ± 7	22 ± 7∗	18 ± 7∗	<0.001
Peak VO_2_ (%)	69 ± 21	63 ± 18∗	52 ± 20∗	<0.001
NYHA I	5651 (78)	322 (71)∗	202 (62)∗	<0.001
CHD severity				<0.001
Mild	1925 (27)	80 (18)	76 (23)	
Moderate	4877 (68)	329 (73)	215 (66)	
Complex	401 (6)	42 (9)	36 (11)	
Surgical history				
No. of prior sternotomies	2.2 ± 0.7	3.2 ± 0.8	3.1 ± 0.7	<0.001
Pacemaker implantation	322 (5)	57 (13)	50 (15)	<0.001
Anatomic data				
LVOT disease	1080 (15)	68 (15)∗	66 (20)∗	0.04
Comorbidities				
Hypertension	1757 (24)	135 (30)	118 (36)	<0.001
Coronary artery disease	347 (5)	48 (11)	46 (14)	<0.001
Diabetes	479 (7)	37 (8)	39 (12)	0.002
Hyperlipidemia	1536 (21)	110 (24)	94 (29)	0.003
Chronic kidney disease III-V	287 (4)	35 (8)∗	44 (14)∗	<0.001
Atrial fibrillation	944 (13)	132 (29)	97 (30)	<0.001
Atrial flutter/tachycardia	640 (9)	74 (16)	55 (17)	<0.001
Laboratory indices				
Estimated GFR (mL/min/1.73 m^2^)	95 (78; 111)	89 (72; 105)	81 (55; 81)	<0.001
MELD-XI	9.44 (9.44; 10.78)	9.44 (9.44; 11.78)∗	11.16 (9.44; 14.29)∗	<0.001
NT-proBNP (pg/mL)	182 (77; 508)	410 (147; 1070)∗	768 (282; 2393)∗	<0.001
Medications				
Loop diuretics	1359 (19)	145 (32)∗	148 (45)∗	<0.001
ACEI/ARB/ARNI	1399 (19)	173 (38)∗	149 (46)∗	<0.001
MRA	327 (5)	46 (10)	51 (16)	<0.001
β-Blockers	2161 (30)	198 (44)	163 (50)	<0.001

Data are presented as mean ± standard deviation, median (Q1; Q3), and count (%). Between-group comparisons were based on the Fisher exact test, analysis of variance test, or Wilcoxon rank sum test, as appropriate.

ACEI/ARB/ARNI, angiotensin-converting enzyme inhibitor/angiotensin 2 receptor blocker/angiotensin receptor/neprilysin inhibitor; CHD, congenital heart disease; GFR, glomerular filtration rate; HF, heart failure; LVOT, left ventricular outflow tract; MELD-XI, model for end-stage liver disease excluding international normalized ratio; MRA, mineralocorticoid receptor antagonist; mrEF, midrange ejection fraction; NT-proBNP, N-terminal pro–B-type natriuretic peptide; NYHA, New York Heart Association; pEF, preserved ejection fraction; rEF, reduced ejection fraction; VO_2_, oxygen consumption.

∗ Signifies the statistically significant difference for pairwise comparison between the rEF and mrEF subgroups.

Table 2 compares echocardiography, cardiac magnetic resonance imaging, and cardiac catheterization data across the 3 groups. By design, there was a trend toward a lower LVEF across the 3 groups, as evidenced by an LVEF of 62% ± 5%, 45% ± 3%, and 31% ± 6% for the pEF, HFmrEF, and HFrEF subgroups, respectively (*P* < 0.001). Similarly, there was a trend toward lower LV global longitudinal strain across the 3 groups, as evidenced by an LV global longitudinal strain of –21% ± 3%, –16% ± 3%, and –12% ± 3% for the pEF, HFmrEF, and HFrEF subgroups, respectively (*P* < 0.001) (Table 2). Compared with the pEF subgroup, patients in the HFmrEF and HFrEF groups had worse chamber dilation and dysfunction, and worse invasive hemodynamic indices (Table 2).

**Table 2:** Hemodynamic indices

	pEF (N = 7203, 90%)	HFmrEF (N = 451, 6%)	HFrEF (N = 327, 4%)	*P*
Echocardiographic data				
Systemic indices				
LA volume index (mL/m^2^)	30 ± 14	37 ± 21	41 ± 13	<0.001
LA reservoir strain (mL/m^2^)	34 ± 11	25 ± 10∗	19 ± 11∗	<0.001
LV end-diastolic volume index (mL/m^2^)	51 (42; 62)	57 (45; 73)∗	83 (58; 108)∗	<0.001
LV end-systolic volume index (mL/m^2^)	20 (16; 25)	29 (21; 38)∗	54 (35; 72)∗	<0.001
LV ejection fraction (%)	62 ± 5	45 ± 3∗	31 ± 6∗	<0.001
LV longitudinal strain (%)	–21 ± 3	–16 ± 3∗	–12 ± 3∗	<0.001
≥Moderate mitral regurgitation	297 (4)	30 (7)	39 (12)	<0.001
≥Moderate aortic regurgitation	440 (6)	28 (6)	27 (8)	0.32
Aortic mean gradient (mm Hg)	6 (3; 18)	5 (2; 13)	6 (3; 20)	0.11
Cardiac index (L/min/m^2^)	2.91 (2.52; 3.58)	2.69 (2.31; 3.51)	2.70 (2.34; 3.51)	<0.001
Nonsystemic indices				
RA volume index (mL/m^2^)	34 (33; 49)	45 (31; 73)	43 (31; 71)	<0.001
RA reservoir strain (%)	32 (23; 43)	22 (15; 31)∗	18 (11; 31)∗	<0.001
Mean RA pressure (mm Hg)	5 (5; 10)	5 (5; 10)	5 (5; 15)	<0.001
RV systolic pressure (mm Hg)	33 (28; 43)	38 (30; 49)	43 (34; 59)	<0.001
RV end-diastolic area index (cm^2^)	29 (22; 41)	36 (27; 51)	34 (26; 44)	<0.001
RV end-systolic area index (cm^2^)	18 (13; 27)	23 (17; 37)	23 (16; 35)	<0.001
RV fractional area change (%)	35 (22; 46)	23 (19; 44)	24 (17; 41)	<0.001
RV free wall strain (%)	–25 (21; 28)	–21 (16; 25)∗	–18 (13; 25)∗	<0.001
≥Moderate tricuspid regurgitation	1257 (18)	129 (27)	93 (28)	<0.001
≥Moderate pulmonary regurgitation	873 (12)	73 (17)	51 (16)	0.006
Pulmonary valve mean gradient (mm Hg)	9 (5; 18)	8 (5; 15)	8 (5; 15)	0.40
CMRI data				
Systemic indices				
LV end-diastolic volume index (mL/m^2^)	70 (56; 82)	73 (61; 97)∗	85 (64; 119)∗	<0.001
LV end-systolic volume index (mL/m^2^)	28 (20; 35)	33 (25; 49)∗	57 (29; 81)∗	<0.001
LV ejection fraction (%)	60 (54; 66)	47 (41; 56)∗	36 (22; 45)∗	<0.001
LV stroke volume index (mL/m^2^)	41 (34; 50)	42 (33; 50)	37 (30; 46)	0.12
Nonsystemic indices				
RV end-diastolic volume index (mL/m^2^)	108 (85; 142)	111 (83; 152)	99 (77; 138)	0.27
RV end-systolic volume index (mL/m^2^)	56 (40; 78)	62 (41; 92)	55 (37; 91)	0.29
RV ejection fraction (%)	49 (41; 55)	44 (36; 52)	44 (30; 50)	<0.001
Cardiac catheterization data				
RA pressure (mm Hg)	8 (6; 12)	11 (8; 15)	12 (8; 17)	<0.001
PA mean pressure (mm Hg)	22 (17; 30)	24 (19; 37)∗	28 (222; 39)∗	<0.001
PA wedge pressure (mm Hg)	12 (9; 16)	14 (10; 18)∗	16 (12; 21)∗	<0.001
Cardiac index (L/min/m^2^)	2.41 (2.08; 3.12)	2.29 (1.89; 2.94)	2.10 (1.78; 2.59)	0.003
PVR index (WU m^2^)	3.46 (2.07; 5.79)	5.31 (3.12; 7.98)	4.49 (3.07; 7.99)	0.004

Data are presented as mean ± standard deviation, median (Q1; Q3), and count (%). Between-group comparisons were based on the Fisher exact test, analysis of variance test, or Wilcoxon rank sum test, as appropriate.

CMRI, cardiac magnetic resonance imaging; HF, heart failure; LA, left atrium; LV, left ventricle; mrEF, midrange ejection fraction; PA, pulmonary artery; pEF, preserved ejection fraction; PVR, pulmonary vascular resistance; RA, right atrium; rEF, reduced ejection fraction; RV, right ventricle.

∗ Signifies the statistically significant difference for pairwise comparison between the rEF and mrEF subgroups.

Of 7981 patients, 4229 (53%) had isolated right heart disease. The proportion of patients with HFrEF was similar in patients with isolated right heart disease compared with others (4% [167 of 4229] vs 4% [160 of 3752], *P* = 0.48). However, the proportion of patients with HFrEF was higher in patients with LVOT disease compared with others (6% [66 of 1214] vs 4% [261 of 6767], *P* = 0.01).

### HF stage

Of the 327 patients in the HFrEF group, 202 (62%) had stage B HF (NYHA class I), whereas 125 (38%) had stage C HF (NYHA II/III). Of the 451 patients in the HFmrEF group, 322 (71%) had stage B HF (NYHA I), whereas 129 (29%) had stage C HF (NYHA II/III). The HFrEF group had a higher prevalence of stage C HF compared with the HFmrEF group (*P* = 0.004). The prevalence of coronary artery disease was similar between the HFrEF and HFmrEF groups (14% vs 11%, *P* = 0.15).

### Correlates of HFrEF

Table 3 shows univariable model of the correlates of HFrEF. On multivariable analysis, the correlates of rEF were older age (odds ratio [OR]: 1.10, 95% confidence interval [CI]: [1.06; 1.14] per 5 years, *P* < 0.001), male sex (OR: 1.70 [95% CI: 1.35; 2.15], *P* < 0.001), pacemaker implantation (OR: 2.62 [95% CI: 2.01; 3.41], *P* < 0.001), number of prior sternotomies (OR: 1.16 [95% CI: 1.05; 1.38], *P* = 0.01), hypertension (OR: 1.72 [95% CI: 134; 2.17], *P* < 0.001), coronary artery disease (OR: 2.97 [95% CI: 2.13; 4.14], *P* < 0.001), and atrial fibrillation (OR: 1.58 [95% CI: 1.12; 2.69], *P* = .009) (Table 4). Of note, CHD severity was not associated with HFrEF on multivariable analysis. Stratified analysis showed that the correlates of HFrEF were similar in males, females, and in patients with isolated right heart disease (Table 4).

**Table 3:** Univariable logistic regression models showing correlates of HFrEF

	Univariable analysis	
	OR (95% CI)	*P*
Demographic indices		
Age (per 5 y)	1.13 (1.09; 1.17)	<0.001
Male sex	1.78 (1.42; 2.24)	<0.001
Body mass index (kg/m^2^)	1.01 (0.99; 1.02)	0.09
CHD severity		
Simple	Reference	
Moderate	1.09 (0.84; 1.43)	0.52
Complex	2.14 (1.41; 3.21)	0.001
Anatomic data		
LVOT disease	1.86 (1.23; 2.69)	0.004
Isolated RH disease	0.92 (0.73; 1.15)	0.47
Surgical history		
Pacemakers	3.42 (2.65; 4.38)	<0.001
No. of prior sternotomies	1.28 (1.11; 1.48)	0.001
Comorbidities		
Hypertension	1.72 (1.36; 2.17)	<0.001
Coronary artery disease	2.97 (2.13; 4.14)	<0.001
Diabetes	1.87 (1.32; 2.65)	<0.001
Atrial fibrillation	2.58 (2.02; 3.29)	<0.001
Atrial flutter/tachycardia	1.97 (1.46; 2.65)	<0.001

LVOT disease and isolated RH disease were modeled as binary variables (yes vs no).

CI, confidence interval; CHD, congenital heart disease; HFrEF, heart failure with reduced ejection fraction; LVOT, left ventricular outflow tract; OR, odds ratio; RH, right heart.

**Table 4:** Multivariable logistic regression models showing correlates of HFrEF

	All patients (N = 7981)		Males (N = 3870, 48%)		Females (N = 4111, 52%)		Isolated RH disease (N = 4229, 53%)	
	OR (95% CI)	*P*	OR (95% CI)	*P*	OR (95% CI)	*P*	OR (95% CI)	*P*
Demographic indices								
Age (per 5 y)	1.10 (1.06; 1.14)	<0.001	1.12 (1.07; 1.18)	<0.001	1.09 (1.03; 1.15)	0.008	1.09 (1.03; 1.15)	0.004
Male sex	1.70 (1.35; 2.15)	<0.001	N/A		N/A		1.77 (1.26; 2.50)	0.001
Surgical history								
Pacemakers	2.62 (2.01; 3.41)	<0.001	2.49 (1.76; 3.51)	<0.001	2.59 (1.66; 3.95)	<0.001	2.72 (1.85; 3.97)	<0.001
No. of prior sternotomies	1.16 (1.05; 1.38)	0.01	1.71 (1.16; 2.54)	0.009	1.49 (1.18; 1.89)	<0.001	1.36 (1.09; 1.68)	0.008
Anatomic data								
LVOT disease	–	–	–	–	1.66 (1.19; 2.73)	0.008	–	–
Comorbidities								
Hypertension	1.72 (1.36; 2.17)	<0.001	1.68 (1.25; 2.12)	0.007	–	–	–	–
CAD	2.97 (2.13; 4.14)	<0.001	1.57 (1.02; 2.43)	0.03	2.62 (1.11; 3.7)	0.002	2.13 (1.33; 3.42)	0.002
Diabetes	1.87 (1.32; 2.65)	<0.001	–	–	2.39 (1.11; 5.48)	0.02	–	–
Atrial fibrillation	1.58 (1.12; 2.69)	0.009	1.45 (1.03; 2.51)	0.01	1.63 (1.18; 2.42)	0.004	1.51 (1.01; 2.31)	0.04

Covariates used in the multivariable models were derived from univariable analysis (see Table 3), and final covariate selection was based on stepwise backward selection with *P* < 0.01 required for a covariate to remain in the model.

"–" signifies covariates with *P* ≥ 0.1.

CAD, coronary artery disease; CI, confidence interval; HFrEF, heart failure with reduced ejection fraction; LVEF, left ventricular ejection fraction; LVOT, left ventricular outflow tract; N/A, not applicable; OR, odds ratio; RH, right heart.

### Prognostic implications of reduced LVEF

**Figure 1:**
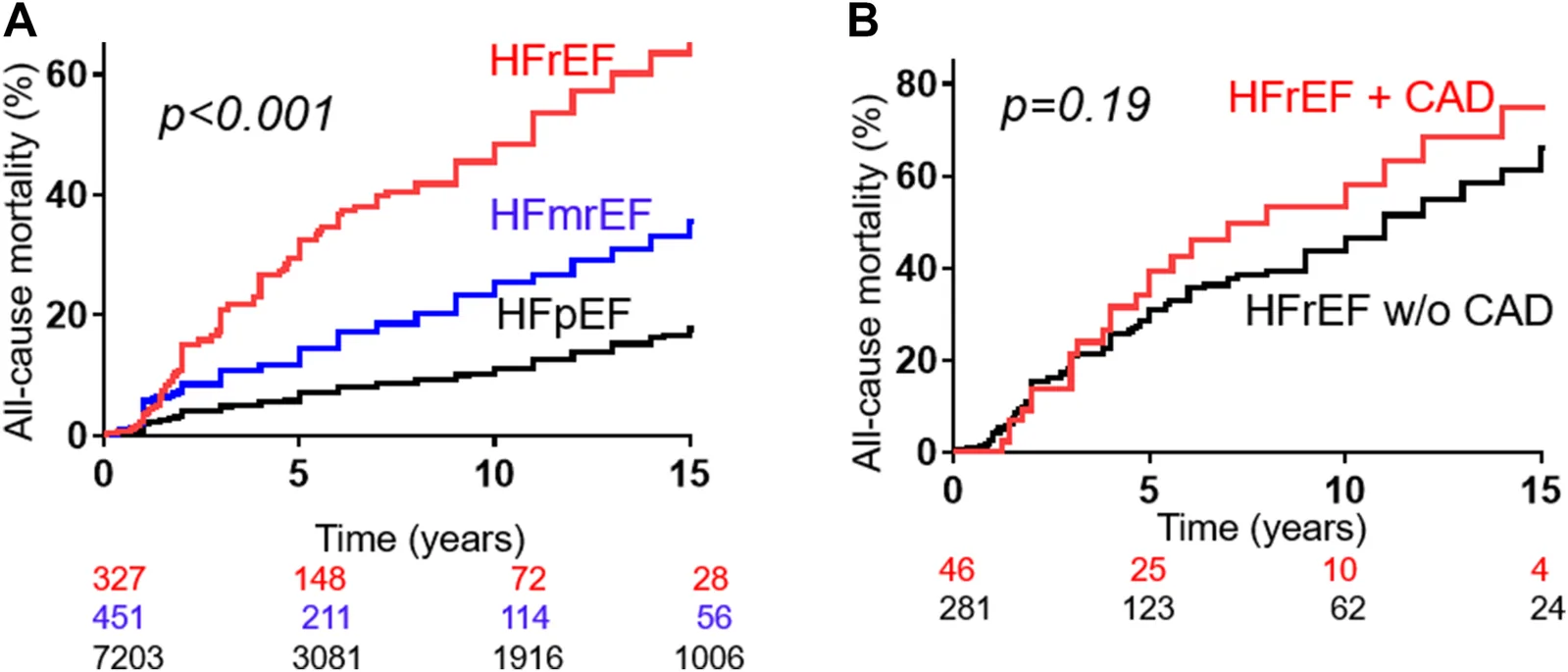
Kaplan-Meier analysis comparing cumulative incidence of all-cause mortality. (**A**) The incidence of mortality was significantly different in patients with heart failure with reduced ejection fraction (HFrEF) (**red**) vs HF with midrange EF (HFmrEF) (**blue**) vs HF with preserved EF (HFpEF) (**black**). (**B**) The incidence of mortality was similar in patients with HFrEF and coronary artery disease (CAD) (**red**) vs HFrEF without CAD (**black**).

Of 7981 patients, 818 (10%) died during follow-up, and the 15-year cumulative incidence of all-cause mortality was 18%, 36%, and 67% in the pEF, HFmrEF, and HFrEF groups, respectively (*P* < 0.001) (Fig. 1). Within the HFrEF group, there was no significant difference in the 15-year cumulative incidence of all-cause mortality between patients with and without coronary artery disease (74% vs 61%, respectively, *P* = 0.19) (Fig. 1).

Table 5 shows the univariable model of correlates of all-cause mortality. On multivariable analysis, having HFmrEF was associated with a 64% increase in all-cause mortality (adjusted hazard ratio [HR]: 1.64 [95% CI: 1.29; 2.09], *P* < 0.001), whereas HFrEF was associated with more than a 3-fold increase in all-cause mortality (adjusted HR: 3.33 [95% CI: 2.72; 4.27], *P* < 0.001) compared with pEF after adjustment for demographic indices, CHD severity, surgical history, anatomic data, laboratory indices, and comorbidities (Table 6).

**Table 5:** Univariable Cox regression model assessing correlates of all-cause mortality

	HR (95% CI)	*P*
LV systolic function		
pEF	Reference	
HFmrEF	2.28 (1.82; 2.86)	<0.001
HFrEF	5.10 (4.22; 6.16)	<0.001
Other echo indices		
RV fractional area change (per 5%)	0.93 (0.91; 0.96)	<0.001
≥Moderate tricuspid regurgitation	1.87 (1.60; 2.19)	<0.001
≥Moderate mitral regurgitation	2.22 (1.77; 2.79)	<0.001
Demographic indices		
Age (per 5 y)	1.23 (1.20; 1.25)	<0.001
Male sex	1.09 (0.95; 1.25)	0.23
Body mass index (per 5 kg/m^2^)	1.06 (1.03; 1.09)	<0.001
CHD severity		
Simple	Reference	
Moderate	1.26 (1.06; 1.50)	0.009
Complex	2.16 (1.65; 2.78)	<0.001
Surgical history		
Pacemakers	1.56 (1.31; 1.85)	<0.001
No. of prior sternotomies	1.38 (1.27; 1.51)	<0.001
Anatomic data		
LVOT disease	1.86 (1.49; 2.29)	<0.001
Laboratory indices		
GFR (per 10 mL/min/1.73 m^2^)	0.77 (0.75; 0.79)	<0.001
MELD-XI	1.15 (1.13; 1.16)	<0.001
Log NT-proBNP (pg/mL)	1.70 (1.61; 1.79)	<0.001
Comorbidities		
Hypertension	1.79 (1.55; 2.06)	<0.001
Coronary artery disease	2.66 (2.19; 3.24)	<0.001
Diabetes	2.36 (1.96; 2.86)	<0.001
Atrial fibrillation	2.69 (2.31; 3.12)	<0.001
Atrial flutter/tachycardia	1.39 (1.14; 1.70)	0.002
Biomarkers		
Log NT-proBNP (pg/mL)	1.70 (1.61; 1.79)	<0.001
Invasive hemodynamic indices		
RA mean pressure (mm Hg)	1.05 (1.04; 1.06)	<0.001
PA mean pressure (mm Hg)	1.03 (1.02; 1.04)	<0.001
PA wedge pressure (mm Hg)	1.03 (1.02; 1.04)	<0.001
PVR index (WU m^2^)	1.03 (1.01; 1.05)	0.008
Cardiac index (L/min/m^2^)	0.89 (0.76; 1.05)	0.20

LVOT disease was modeled as a binary variable (yes vs no).

CHD, congenital heart disease; CI, confidence interval; GFR, glomerular filtration rate; HF, heart failure; HR, hazard ratio; LVOT, left ventricular outflow tract; MELD-XI, model for end-stage liver disease excluding international normalized ratio; mrEF, midrange ejection fraction; NT-proBNP, N-terminal pro–B-type natriuretic peptide; PA, pulmonary artery; pEF, preserved ejection fraction; PVR, pulmonary vascular resistance; RA, right atrium; rEF, reduced ejection fraction.

**Table 6:** Multivariable Cox regression model assessing prognostic implication of rEF in different patient subgroups

	All patients (N = 7981)		Patients with NT-proBNP (N = 3838)		Patients with catheterization data (N = 1710)	
	HR (95% CI)	*P*	HR (95% CI)	*P*	HR (95% CI)	*P*
LV systolic function						
pEF	Reference		Reference		Reference	
mrEF	1.64 (1.29; 2.09)	<0.001	1.70 (1.22; 2.36)	0.001	1.32 (0.68; 2.55)	0.41
rEF	3.33 (2.72; 4.27)	<0.001	2.99 (2.30; 3.91)	<0.001	1.93 (1.20; 3.09)	0.007
Other echo indices						
RV FAC, per 5%	0.93 (0.90; 0.97)	0.01	–	–	–	–
≥Moderate tricuspid regurgitation	1.32 (1.12; 1.56)	0.001	1.32 (1.03; 1.69)	0.03	–	–
≥Moderate mitral regurgitation	1.47 (1.15; 1.86)	0.002	–	–	–	–
Demographic indices						
Age (per 5 y)	1.22 (1.18; 1.27)	0.002	1.16 (1.12; 1.19)	<0.001	1.18 (1.12; 1.25)	<0.001
Body mass index (per 5 kg/m^2^)	1.06 (1.02; 1.10)	0.003	–	–	–	–
CHD severity						
Simple	Reference					
Moderate	1.02 (0.80; 1.29)	0.89	–	–	–	–
Complex	1.82 (1.22; 2.74)	0.004	–	–	–	–
Surgical history						
No. of prior sternotomies	1.42 (1.24; 1.62)	<0.001	–	–	–	–
Anatomic data						
LVOT disease	–	–	–	–	2.63 (1.61; 4.31)	<0.001
Laboratory indices						
MELD-XI	–	–	1.07 (1.05; 1.09)	<0.001	1.09 (1.06; 1.12)	<0.001
Comorbidities						
Diabetes	1.51 (1.234; 1.84)	<0.001	1.59 (1.23; 2.05)	<0.001	–	–
Atrial fibrillation	1.32 (1.12; 1.56)	<0.001	–	–	–	–
Biomarkers						
Log NT-proBNP (pg/mL)			1.53 (1.44; 1.62)	<0.001		
Invasive hemodynamic indices						
RA mean pressure (mm Hg)					1.04 (1.02; 1.06)	<0.001
PA mean pressure (mm Hg)					1.03 (1.02; 1.04)	<0.001

Covariates with *P* < 0.1 on univariable analysis (Table 5) were used to create the multivariable model, and the final covariate selection was based on stepwise backward selection with *P* < 0.1 required for a covariate to remain in the multivariable model.

"–" signifies covariates with *P* ≥ 0.1. LVOT disease defined as having any of the following conditions: aortic valve prosthesis, subvalvular, valvular, or supravalvular aortic stenosis (mean gradient >20 mm Hg) or ≥moderate aortic regurgitation, in the absence of mitral valve disease.

CHD, congenital heart disease; CI, confidence interval; FAC, fractional area change right ventricle; HR, hazard ratio; LVOT, left ventricular outflow tract; MELD-XI, model for end-stage liver disease excluding international normalized ratio; mrEF, midrange ejection fraction; NT-proBNP, N-terminal pro–B-type natriuretic peptide; PA, pulmonary artery; pEF, preserved ejection fraction; RA, right atrium; rEF, reduced ejection fraction; RV, right ventricular.

On subgroup analysis of patients with NT-proBNP data (N = 3838), HFmrEF (adjusted HR: 1.70 [95% CI: 1.22; 2.39], *P* = 0.001) and HFrEF (adjusted HR: 2.99 [95% CI: 2.30; 3.91], *P* < 0.001), as well as NT-proBNP (adjusted HR: 1.53 [95% CI: 1.44; 1.62] per 1 standard deviation increment, *P* < 0.001), were associated with all-cause mortality (Table 6). Similarly, on subgroup analysis of patients with cardiac catheterization data (N = 1710), HFrEF (adjusted HR: 1.93 [95% CI: 1.20; 3.09], *P* = 0.007), as well as right atrial mean pressure (adjusted HR: 1.04 [95% CI: 1.02; 1.05] per 1 mm Hg increment, *P* < 0.001) and pulmonary artery mean pressure (adjusted HR: 1.03 [95% CI: 1.02; 1.04] per 1 mm Hg increment, *P* < 0.001), was associated with all-cause mortality (Table 6).

## Discussion

In this study, we assessed the prevalence, predictors, and prognostic implications of LV systolic dysfunction among 7981 adult CHD patients with morphologic LV as the systemic ventricle. The main findings are as follows: (1) approximately 10% of the cohort had LV systolic dysfunction, of which 6% had HFmrEF and 4% had HFrEF; (2) the correlates of HFrEF were older age, male sex, pacemaker implantation, multiple prior sternotomies, and acquired comorbidities such as hypertension, coronary artery disease, diabetes, and atrial fibrillation; and (3) LV systolic dysfunction (both HFmrEF and HFrEF) was associated with all-cause mortality.

HF is a growing public health and economic problem in the United States because of an aging population and rising prevalence of comorbidities.^12^^,^^20^ Among patients with HF, those with HFrEF have increased risk of mortality compared with the other HF categories, underscoring the importance of LVEF in outcomes among patients with HF.^12^^,^^20^ The current study demonstrates a similar pattern of outcomes observed in the acquired heart disease population, with a 3-fold increase in mortality among patients with HFrEF compared with patients with pEF. Furthermore, we observed an increased risk of mortality among patients with HFmrEF relative to the pEF group. In the acquired heart disease population, HFmrEF is a poorly characterized subgroup with a natural history that is better than HFrEF but worse than that of patients with pEF. This is reflected in the guideline recommendations, whereby the use of GDMT is class II recommendation in patients with HFmrEF compared with class I recommendation in patients with HFrEF.^12^ The current study demonstrates significant risk of mortality in patients with HFmrEF as well as HFrEF and suggests that any degree of reduction in LVEF carries significant negative prognostic implications in this population. A noteworthy finding was that CHD severity, which is a known determinant of outcomes in this population, was not a predictor of mortality in our model after adjustment for the LVEF-based HF category.^8^^,^^10^^,^^11^ We postulate that patients with moderate and complex CHD had a higher prevalence of risk factors for LV systolic dysfunction (such as multiple sternotomies, pacemaker implantation, and atrial fibrillation), and that the elevated risk of mortality previously reported in patients with moderate and complex CHD was likely related to LV systolic dysfunction in this subgroup of patients.

Most of the predictors (correlates) of HFrEF observed in this study (older age, male sex, pacemaker implantation, and comorbidities) are consistent with risk factors previously described in the acquired heart disease population.^12^^,^^20^ An important novel finding was the association between number of prior sternotomies and prevalence of HFrEF. We postulate that this may be related to myocardial injury resulting from cardiopulmonary bypass, and the patients with multiple reoperations were exposed to repeated episodes of myocardial injury.^21^^,^^22^ The cumulative effect of these events resulted in LV systolic dysfunction.

### Clinical implications and future directions

The use of GDMT (β-blockers, renin angiotensin system antagonist, mineralocorticoid receptor antagonist, and sodium-glucose cotransporter 2 inhibitor) is a class I recommendation in patients with HFrEF due to acquired heart disease.^12^ Although patients with CHD were not included in the clinical trials on which these guideline recommendations were based, emerging data in the CHD literature suggests that these therapies are effective in this population.^4^^,^^23^ In a recent study, Neijenhuis et al.^24^ demonstrated that patients with CHD who were already on GDMT had a 3-fold reduction in HF hospitalization after the initiation of sodium-glucose cotransporter-2 inhibitor. Similarly, Nederend et al.^25^ and Fusco et al.^26^^,^^27^ reported improvement in systemic ventricular EF, 6-minute walk distance, and congestion (lower NT proBNP level) after initiation of GDMT. This emerging body of evidence, coupled with the negative prognostic implication of HF in CHD, underscores the urgent need for HF therapies in CHD.

### Limitations

We did not have invasive coronary angiogram or coronary computed tomography angiography in all patients with LV systolic dysfunction and were therefore unable to objectively determine what proportion of these patients had ischemic cardiomyopathy. In addition, we did not have data about temporal changes in the use of GDMT or compliance with GDMT, and hence, we were unable to assess the effect of medical therapy on LVEF and mortality.

## Conclusions

Approximately 10% of adults with CHD had LV systolic dysfunction (HFrEF and HFmrEF), and these patients had an increased risk of mortality compared with patients with pEF. These data suggest that any degree of LV systolic dysfunction had a detrimental effect on the long-term outcome of patients with CHD. Given the elevated risk of mortality in patients with LV systolic dysfunction (both mrEF and rEF), and the emerging data about the efficacy of GDMT in patients with CHD, there is an urgent need to implement GDMT in this population. Further studies are required to assess the impact of GDMT on the long-term survival in this population.

## Ethics Statement

The research reported in this paper adhered to STROBE Statement (STrengthening the Reporting of OBservational studies in Epidemiology) guidelines.

## Patient Consent

The authors confirm that patient consent is not applicable to this article. This is a retrospective case report using deidentified data, and therefore the institutional review board did not require consent from the patient.

## Funding Sources

ACE is supported by 10.13039/100000050National Heart, Lung, and Blood Institute grants (R01 HL158517, R01 HL160761, and R01 HL162830). The MACHD Registry is supported by the Al-Bahar Research grant.

## Disclosures

The authors have no conflicts of interest to disclose.
