# Supplementary material for: Prognostic Implication of Heart Failure With Reduced and Midrange Ejection Fraction in Adults With Congenital Heart Disease
Source: CJC Pediatr Congenit Heart Dis. 2026 Mar 4;5(4):258–66. doi: 10.1016/j.cjcpc.2026.02.006 (PMC13523820; doi:10.1016/j.cjcpc.2026.02.006)
Supplement: Supplementary file 1 [file mmc1.docx]

**Supplemental Table S1: CHD Severity Groups**

**Simple CHD**

Atrial septal defect

Ventricular septal defect

Patent ductus arteriosus

Pulmonary valve stenosis

**Moderately complex CHD**

Tetralogy of Fallot

Ebstein

Double chamber RV

Coarctation of aorta

Mitral stenosis

Subaortic stenosis

Aortic valve stenosis

Supravalvular AS

PAPVR

Double chamber RV

Cor triatriatum

**Complex CHD**

Pulmonary atresia -IVS

Truncus arteriosus

TGA- arterial switch op

TGA- Rastelli op

Double outlet RV

d-TGA status post atrial switch operation

Congenitally corrected TGA

Fontan physiology

Unrepaired/palliative single ventricle

**Abbreviations**: AS: Aortic stenosis; CHD: Congenital heart disease; IVS: Intact ventricular septum; RV: Right ventricle; TGA: Transposition of great arteries; PAPVR: Partial anomalous pulmonary venous return.

**Supplemental Table S2: CHD Diagnoses**

| **CHD Diagnosis** | **All**  **(N=7981)** | **pEF**  **(N=7203, 90%)** | **mrEF**  **(N=451, 6%)** | **rEF**  **(N=327, 4%)** |
| --- | --- | --- | --- | --- |
| Tetralogy of Fallot | 1225 | 1055 (15%) | 107 (24%) | 63 (19%) |
| Ebstein | 863 | 759 (11%) | 76 (17%) | 28 (9%) |
| Pulm valve stenosis | 466 | 432 (6%) | 19 (4%) | 15 (5%) |
| Pulmonary atresia -IVS | 59 | 51 (0.7%) | 4 (0.9%) | 4 (1.1%) |
| Double chamber RV | 56 | 56 (0.8%) | 0 | 0 |
| Truncus arteriosus | 62 | 52 (0.7%) | 7 (1.6%) | 3 (0.9%) |
| TGA- arterial switch op | 124 | 109 (1.5%) | 9 (2%) | 6 (1.8%) |
| TGA- Rastelli op | 38 | 32 (0.4%) | 3 (0.7%) | 3 (0.9%) |
| Double outlet RV | 58 | 49 (0.7%) | 3 (0.7%) | 6(1.8%) |
| Coarctation of aorta | 985 | 897 (13%) | 44 (10%) | 44 (14%) |
| Mitral stenosis | 19 | 18 (0.3%) | 1 (0.2%) | 0 |
| Subaortic stenosis | 223 | 213 (3%) | 6 (1.3%) | 4 (1.2%) |
| Aortic valve stenosis | 962 | 890 (12%) | 35 (8%) | 37 (11%) |
| Supravalvular AS | 22 | 21 (0.3%) | 1 (0.2%) | 0 |
| PAPVR | 587 | 526 (7%) | 37 (8%) | 24 (11%) |
| Atrial septal defect | 973 | 906 (13%) | 34 (8%) | 33 (10%) |
| Ventricular septal defect | 656 | 580 (8%) | 451 (9%) | 327 (11%) |
| Patent ductus arteriosus | 443 | 411 (6%) | 19 (4%) | 13 (4%) |
| Cor triatriatum | 39 | 34 (0.5%) | 3 (0.7%) | 2 (0.6%) |

**Abbreviations**: AS: Aortic stenosis; CHD: Congenital heart disease; rEF: Reduced ejection fraction; mrEF: Mid-range ejection fraction; pEF: Preserved ejection fraction; IVS: Intact ventricular septum; RV: Right ventricle; TGA: Transposition of great arteries; PAPVR: Partial anomalous pulmonary venous return.
